# Anti-cancerous effect of cis-khellactone from *Angelica amurensis* through the induction of three programmed cell deaths

**DOI:** 10.18632/oncotarget.24686

**Published:** 2018-03-30

**Authors:** Samil Jung, Hyung-In Moon, Beom Suk Lee, Subeen Kim, Nguyen Thi Ngoc Quynh, Jimin Yu, Dan-Diem Thi Le, Zolzaya Sandag, Hyegyeong Lee, Hyojeong Lee, Nguyen Hai Anh, Young Yang, Jong-Seok Lim, Keun-Il Kim, Myeong-Sok Lee

**Affiliations:** ^1^ Department of Biological Science, Sookmyung Women's University, Seoul, 14310, South Korea; ^2^ Department of Medicinal Biotechnology, College of Health Sciences, Dong-A University, Busan, 49315, South Korea

**Keywords:** Angelica amurensis, cis-khellactone, anti-cancer drug, apoptosis, autophagy-mediated cell death, necrosis/necroptosis

## Abstract

*Angelica amurensis* has traditionally been used to treat various medical problems. In this report, we introduce cis-khellactone as a new anti-cancer agent, which was isolated from the chloroform soluble fraction of the rhizomes of *Angelica amurensis*. Its anti-cancerous effect was at first tested in MCF7 and MDA-MB-231 breast cell lines, in which MCF7 is well known to be resistant to many anti-cancer drugs; MCF10A normal breast cell line was used as a control. *In vitro* experiments showed that cis-khellactone suppressed cell growth and proliferation at a relatively low concentrations (<5 μg/ml) and decreased cell viability at high concentrations (>10 μg/ml) in both cancer cell lines in a time- and concentration-dependent manner. This anti-cancerous effect was also checked in additional 16 different types of normal and cancer cell lines. Cis-khellactone treatment significantly suppressed cell proliferation and enhanced cell death in all tested cancer cell lines. Furthermore, Western blot analysis showed that cis-khellactone induced three types of programmed cell death (PCD): apoptosis, autophagy-mediated cell death, and necrosis/necroptosis. Cis-khellactone concentration-dependently decreased cell viability by increasing the level of reactive oxygen species (ROS) and decreasing mitochondrial membrane potential (MMP), which are related to all three types of PCD. Mitochondrial fractionation data revealed that cis-khellactone induced the translocation of BAX and BAK into mitochondria as well as the overexpression of VDAC1, which probably accelerates MMP disruption and finally cell death. Importantly, our extended *in vivo* studies with xenograft model further confirmed these findings of anti-cancerous effects and showed no harmful effects in normal tissues, suggesting that there would be no side effects in humans.

## INTRODUCTION

Despite advances in cancer treatment, it remains a critical health issue for people worldwide. Cancer treatments primarily include surgery, radiation therapy, hormone therapy, and chemotherapy. Chemotherapy uses one or more anti-cancer drugs to directly cure cancer or to reduce symptoms. Traditional chemotherapeutic agents are cytotoxic to cancer cells, interfering with cell division (mitosis), dysregulating cellular metabolism, or inhibiting biosynthesis of nucleic acids or proteins. However, many of these drugs have adverse effects.

A significant issue with chemotherapy is the resistance of cancer cells to many anti-cancer drugs. Different types of cancer cells show varying levels of susceptibility to anti-cancer drugs and even resistance toward one or more types of cell death. Cancer cells promote and aggravate tumorigenesis by increasing resistance to cell death. For years, much effort has been focused on characterizing apoptosis in cancer research, and most of the currently available anti-cancer drugs are designed to trigger apoptosis in tumor cells. However, many malignant cells have acquired strong resistance to these anti-cancer drugs, owing to defects in their apoptotic machinery (e.g., defective p53 or absence of caspases). One strategy to overcome this resistance is to target different types of cell deaths. For a long time, cell death has traditionally been divided into two types: programmed cell death (apoptosis) and non-programmed accidental cell death (necrosis). However, our understanding of cell death has been changed dramatically. Eukaryotic cells are now thought to undergo different types of programmed cell death (PCD) through multiple signaling pathways, depending upon different stimuli and environmental factors. PCD is now divided into three main types: Type I, caspase-dependent apoptosis; Type II, autophagy-mediated cell death; and Type III/IV, caspase-independent necrosis/necroptosis [1–6]. Apoptosis can occur via either the death receptor-mediated *extrinsic pathway* or the mitochondria-mediated *intrinsic pathway.* Autophagy can promote both cell survival and death, although its dual role in cancer remains unclear. Autophagy-mediated cell death uses autophagic machinery that is used for cell survival to induce cell death [7–13]. Necrosis is a form of programmed necrotic cell death mediated by receptor-interacting protein 1 and 3 (RIP1 and RIP3) kinases [14–21]. Necrosis has long been considered to be a non-programmed cell death; however, emerging evidences suggest that necrosis can also be a kind of PCD. Therefore, a new type PCD, necroptosis, was proposed by Xin Teng [14]. Many recent studies have suggested that these three PCD pathways are interconnected [6][22]. Thus, our aim has been to discover new anti-cancer drugs that can induce all three types of PCD in cancer cells.

Another major issue with chemotherapeutic agents is their toxicity to normal tissues. Many currently available anti-cancer drugs are synthetic chemical compounds that can cause long-lasting adverse effects in humans. Thus, effective anti-cancerous agents that have fewer toxic side effects than those presently available are highly sought after. Plant extracts have gained considerable attention as a new source of anti-cancer drugs, and numerous research groups have studied traditional medicinal plants. Thus, we sought to find natural compound that selectively kill only tumor cells without harming normal cells.

This present study aimed to discover a new harmless anti-cancer drug that can trigger more than one type of PCD in cancer cells. For this purpose, we initially focused on cis-khellactone from the chloroform soluble fraction of the rhizomes of *Angelica amurensis*. *Angelica amurensis* has been used as a traditional herbal medicine for the treatment and alleviation of various illnesses and cis-khellactone derivatives have been reported to exhibit a variety of biological effects for the treatment of AIDS, diabetes, malaria and other diseases [23–27].

**Figure 1:**
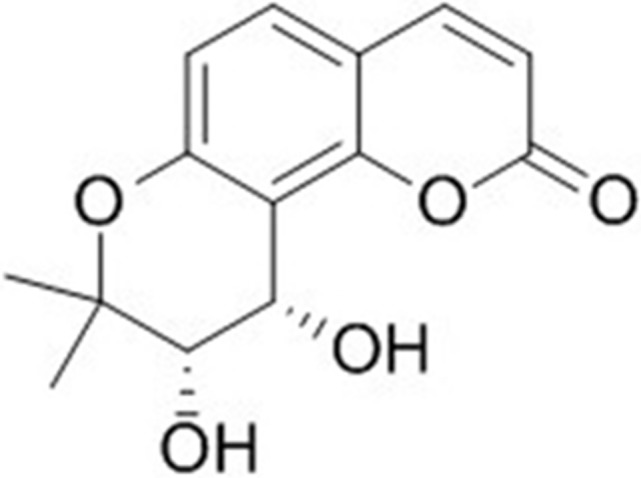
The molecular structure of cis-khellactone

In this study, we found that cis-khellactone (Figure 1) possesses anti-cancerous activity against several different types of cancer cell lines by suppressing cell growth and proliferation or by accelerating three types of PCD (apoptosis, autophagy-mediated cell death, and necrosis/necroptosis).

## RESULTS

### Effects of cis-khellactone on the proliferation and viability of MCF7 and MDA-MB-231 breast cancer cell lines

**Figure 2:**
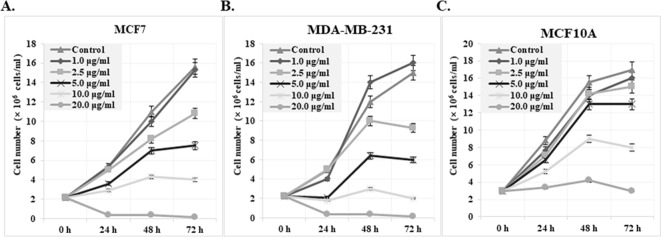
Effect of cis-khellactone on the cell proliferation and viability of MCF7, MDA-MB-231, and MCF10A breast cancer and normal cell lines Growth curve of MCF7 **(A)**, MDAMB-231 **(B)**, and MCF10A **(C)** cells are shown after treatment with indicated concentrations of cis-khellactone. MCF7, MDA-MB-231, and MCF10A cells were treated with DMSO alone (control) or with 1, 2.5, 5, 10 or 20 μg/ml of cis-khellactone for 24, 48, or 72 h. After indicated time periods, the cells were collected and viability was evaluated as described in Materials and Methods. The number represents the viable cells of each cell line.

**Figure 3:**
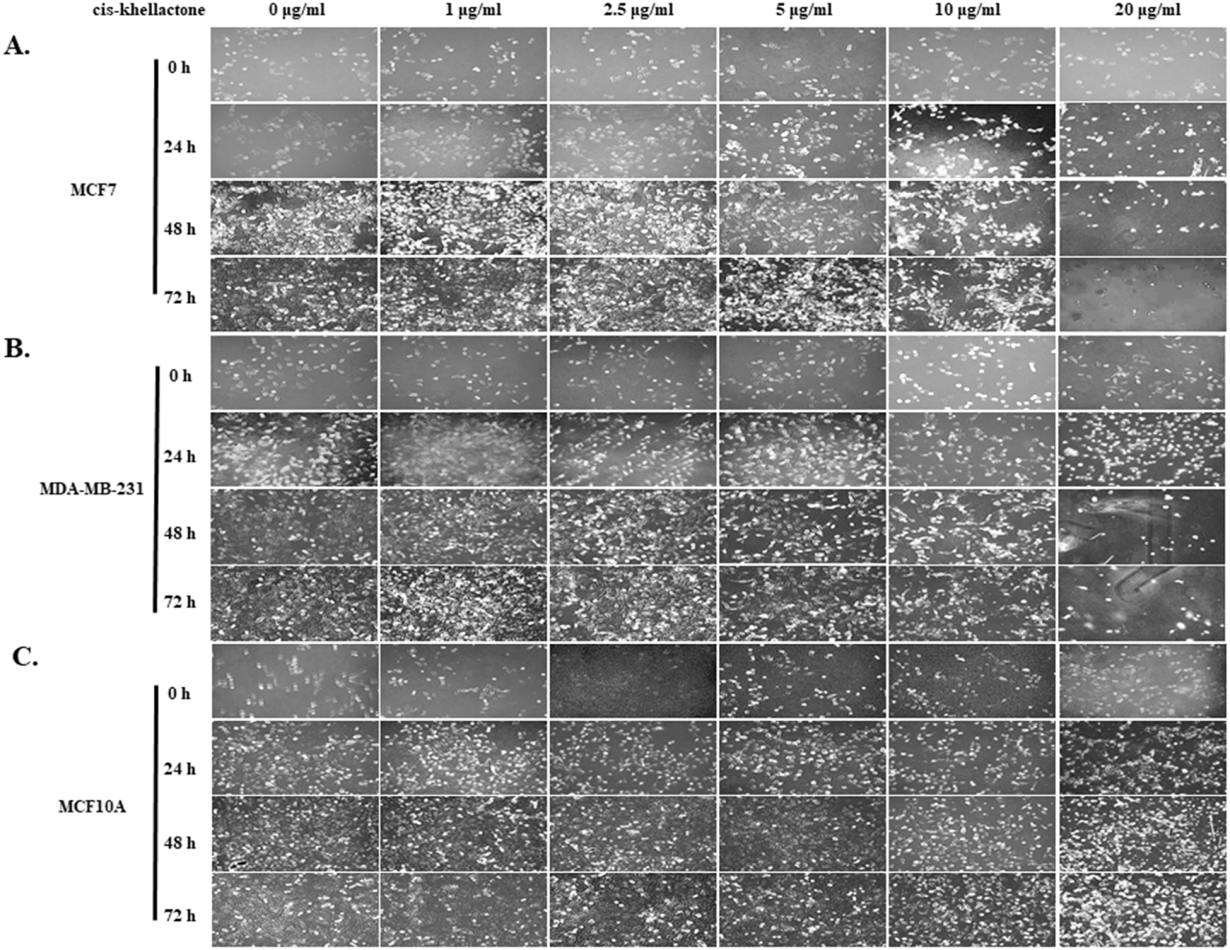
Microscopic morphology of MCF7, MDA-MB-231, and MCF10A cells at various times after treatment with indicated concentrations of cis-khellactone MCF7, MDA-MB-231, and MCF10A cells (2×10^4^) were plated onto 6-well tissue culture dishes and allowed to form a confluent monolayer. Cells were either left untreated (control) or treated with 1, 2.5, 5, 10 or 20 μg/ml of cis-khellactone for 24, 48, or 72 hours. Phenotypes of MCF7 **(A)**, MDA-MB-231 **(B)**, and MCF10A **(C)** cells were photographed under the microscope (Black bar=100 μm). One representative experiment of three is shown.

Cytotoxic activities of cis-khellactone were evaluated by assessing its effects on the proliferation and viability of MCF7 and MDA-MB-231 human breast cancer and MCF10A normal cell lines. In particular, MCF7 was chosen as a good model system to test our hypothesis because it reportedly has a high resistance to many pro-apoptotic anti-cancer drugs; such resistance is probably due to the absence of key proteins (e.g. caspase3 and RIP3) in the processes of apoptosis and necrosis/necroptosis. Briefly, three cell lines were plated onto 24-mm culture dishes and allowed to form a confluent monolayer for 24 h. These cells were then cultured in the absence and presence of various concentrations of cis-khellactone (0, 1, 2.5, 5, 10, 20, 30, 40, 50, or 100 μg/ml) for 0, 24, 48, and 72 h. Morphological changes were first screened under a microscope. Interestingly, cis-khellactone showed a strong cytotoxic effect on MCF7 and MDA-MB-231 cells, but not on MCF10A cells (data not shown). Therefore, we further tested the effects of cis-khellactone on cell growth and morphological changes in a time- and concentration-dependent manner. At a relatively low concentration of cis-khellactone (2.5 or 5 μg/ml), cell growth and proliferation of MCF7 and MDA-MB-231 cells were significantly delayed compared with cells treated with DMSO alone (Figure 2A and 2B). In addition, cell numbers decreased after treatment for 72 h at relatively high concentrations (10 or 20 μg/ml), indicating that cell death was induced (Figure 2A and 2B). These data suggest that cis-khellactone greatly suppressed the proliferation and viability of cancer cells in a time- and concentration-dependent manner. Importantly, MCF10A cells appeared to be much less sensitive to cis-khellactone treatment than the MCF7 and MDA-MB-231 cancer cells, implying that normal cells are less affected by this compound (Figure 2C). It is meaningful that cis-khellactone may be able to inhibit and delay aggressive cancer cell growth and proliferation without adversely affecting normal cells. In a subsequent experiment, microscopic examination confirmed the results of the cell growth data (Figure 3A, 3B, and 3C). Again, this data suggests that cis-khellactone greatly affects cell proliferation and viability in a concentration-dependent manner.

Collectively, our data suggest that cis-khellactone acts as an anti-cancer drug by inhibiting cancer cell proliferation at low concentrations and inducing cell death at high concentrations with minimal toxicity to normal cells.

### Anti-tumor activity of cis-khellactone in various cancer cell lines

**Figure 4:**
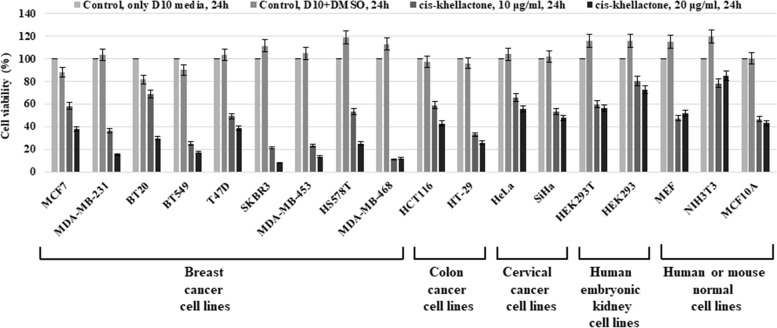
Cell viability, proliferation, and cytotoxicity assay in 18 different types of cancer and normal cell lines after treatment with cis-khellactone Each cell line (2×10^4^/ml) was plated onto 96-well tissue culture dishes for 24 h and then treated either with DMSO alone (control) or with 10 or 20 μg/ml of cis-khellactone for 24 h. Cell viability, proliferation, and cytotoxicity of each cell line were tested as described in Materials and Methods.

The cytotoxic activity of cis-khellactone in MCF7 and MDA-MB-231 cancer cells suggested the possibility of similar effects in other types of cancer cells. Therefore, this investigation was extended to include 16 more different types of normal and cancer cell lines. The cytotoxic effect of cis-khellactone was assessed by using MTT assay after each cell line was treated with either DMSO alone (control) or 10 or 20 μg/ml of cis-khellactone, which was the concentration with the best induction of cell death in the MCF7 and MDA-MB-231 cells. The data revealed significant cytotoxic activity in most of the tested cancer cell lines treated with cis-khellactone (Figure 4). However, normal cells were found to be much less sensitive to cis-khellactone than cancer cell lines (Figure 4).

Taken together, these results suggest that the cis-khellactone has strong anti-tumoral activity and could potentially be used to treat a variety of cancer types.

### Induction of three types of PCD by cis-khellactone in MCF7 and MDA-MB-231 cancer cell lines

**Figure 5:**
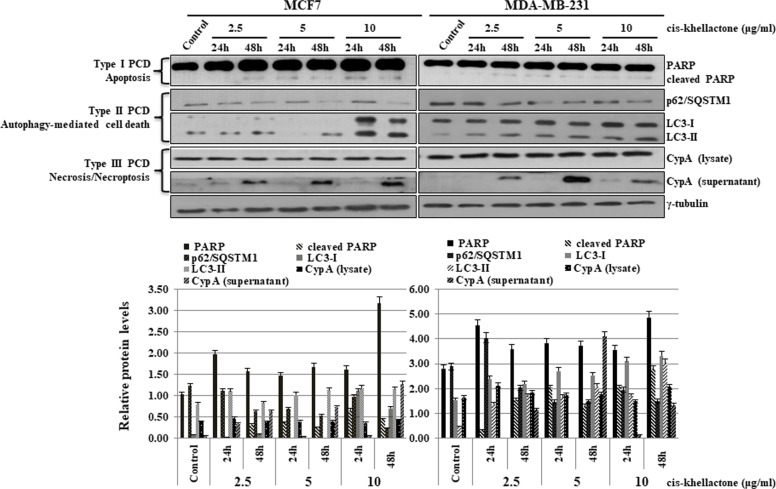
Effect of cis-khellactone on three types of PCD (apoptosis, autophagy-mediated cell death, and necrosis/necroptosis) in MCF7 and MDA-MB-231 cancer cells MCF7 and MDA-MB-231 cells were cultured and treated with 2.5, 5, and 10 μg/ml for 24 or 48 h, while control cells were treated with DMSO alone. Cell lysates were examined by Western blot analysis by using following corresponding biomarkers or regulatory proteins of the different types of cell death; PARP for apoptosis, p62 and LC3 for autophagy, and CypA for necrosis/necroptosis; γ-tubulin was used as an internal control. The result of Western blot was quantified by using ImageJ program.

After determining that cis-khellactone has anti-cancer properties, we next sought to elucidate the underlying mechanism of this effect. We found that concentration of cis-khellactone higher than 10 μg/ml significantly induced cell death. To determine what types of cell death cis-khellactone can induce, both cancer cell lines were treated with either DMSO alone (control) or 2.5, 5, or 10 μg/ml of cis-khellactone for 24 and 48 h. Whole cell lysates were prepared and subjected to the Western blot analysis. Our results showed that cis-khellactone induced all three types of PCD (apoptosis, autophagy-mediated cell death, and necrosis/necroptosis) depending upon the exposure time and concentration. The data showed that cis-khellactone induced autophagy and apoptosis at shorter exposure times, whereas prolonged treatment triggered, all three PCD, especially and mainly necrosis/necroptosis at 48 h (Figure 5). Many studies have reported that autophagy protects cells from cell death in response to apoptosis inducing stresses or anti-cancer drug treatment. Even though autophagy is at first induced for the cell survival, extended and prolonged stresses finally induce autophagy-mediated cell death as well as other types of cell deaths such as apoptosis and necrosis/necroptosis. Our data also revealed similar results. Treatment with cis-khellactone started to decrease p62/SQSTM1 expression levels and to induce the conversion of LC3-I to LC3-II after 24 h, suggesting that autophagy was at first induced probably for the cell survival because no cell death was detected at this time (Figure 5). However, longer treatment of cis-khellactone induced cell death, in which the high level of changes were detected in the p62/SQSTM1 expression levels and LC3 conversion rates. Therefore, we assumed that autophagy was induced at early times (24 h) probably for cell survival and then autophagy-mediated cell death was finally induced at later times. Its treatment also increased the level of cleaved PARP at both 24 and 48 h (Figure 5). Moreover, the export of CypA protein into the extra-cellular environment increased significantly after 48 h of treatment in both cell lines, indicating the induction of necrosis/necroptosis (Figure 5). When both cancer cells were treated with concentrations higher than 10 μg/ml, cell death was significantly induced even after 24 h (data not shown). Altogether, these results strongly suggest that cis-khellactone renders cancer cells more sensitive to three different types of PCD.

In addition, the effect of cis-khellactone on cell migration was also tested by employing a conventional wound healing assay, in which MCF7 and MDA-MB-231 cancer cells were treated with 2.5 or 5 μg/ml cis-khellactone and their migration was found to be inhibited (Supplementary Figure 1).

Taken together, our results showed that cis-khellactone acts as an anti-cancer drug by inhibiting cell growth and migration at low concentration and accelerating three types of PCD (apoptosis, autophagy-mediated cell death, and necrosis/necroptosis) at high concentrations in MDA-MB-231 and MCF7 cancer cells.

### Regulation of three types of PCD by cis-khellactone through the controlling of reactive oxygen species (ROS) and mitochondrial membrane potential (MMP)

**Figure 6:**
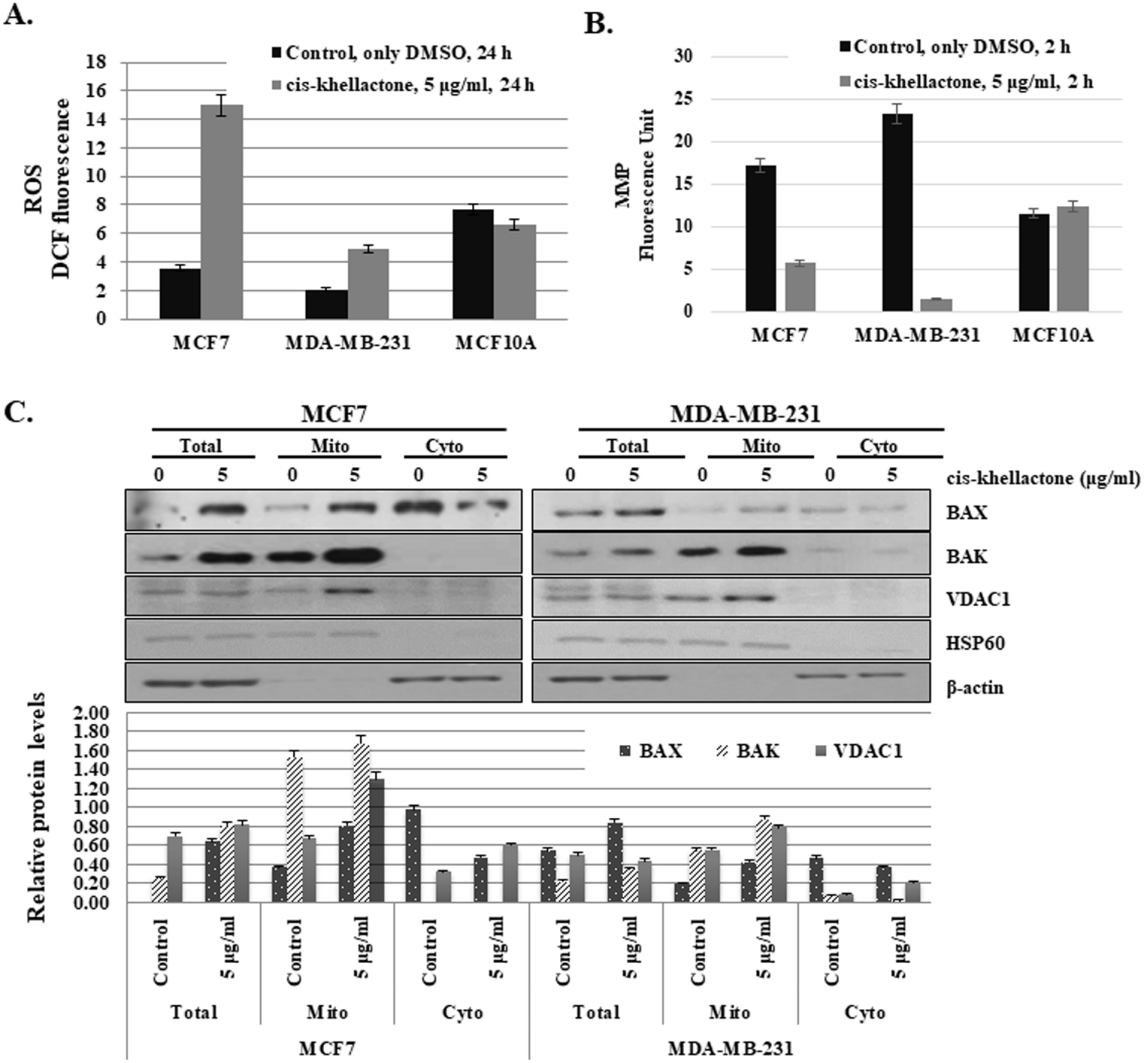
Effect of cis-khellactone on cellular levels of reactive oxygen species (ROS) and mitochondrial membrane potential (MMP) **(A)** Increased ROS production after cis-khellactone treatment in MCF7 and MDA-MB-231 cells. MCF7 and MDA-MB-231 cells were cultured in complete media for 24 h and then treated with 5 μg/ml for 24 h, in which control cells were treated with DMSO alone. Cells were collected and 2.5 × 10^4^ MCF7 cells were plated onto 96-well plates. ROS levels were measured after adding DCFH-DA (see Materials and Methods). The data represent the means ± SD from the three independent experiments (P<0.05). **(B)** Decreased MMP after cis-khellactone treatment in MCF7 and MDA-MB-231 cells. MCF7 and MDA-MB-231 cells were cultured in 96-well tissue culture dishes with DMEM media, followed by treatment with 5 μg/ml of cis-khellactone for 2 h. Mito-ID Membrane Potential Dye Loading Solution was added to each well, and plates were incubated for 30 min at room temperature. MMP was assessed by measuring the resulting fluorescence with a Gemini XPA Microplate Reader. For a positive control, 4 μM carbonyl cyanide 3-chlorophenylhydrazone, an uncoupler of oxidative phosphorylation, was used (see Materials and Methods). The data represent the means ± SD from three independent experiments (P<0.05). **(C)** Mitochondrial fractionation after cis-khellactone treatment. Mitochondrial fractionation was performed as previously described [23], using HSP60 and γ-tubulin as mitochondrial and cytosolic fractionation markers, respectively. The result of Western blot was quantified by using ImageJ program.

The next phase of the experiment was designed to elucidate how cis-khellactone can induce three types of PCD. We initially focused on the effect of cis-khellactone on mitochondrial main functions, regulation of the cellular ROS level and MMP, because eukaryotic mitochondria is known to play vital roles in all three types of PCD [28–30]. In general, cell death inducing many types of stresses (e.g. hypoxia, nutrient starvation, and anti-cancer drugs) increase ROS level and decrease MMP, which make malfunctioned mitochondria and finally stimulate cell death. Mitochondrial metabolism usually produces physiologic ROS to support cell survival; it regulates cellular metabolism, cell signaling, and homeostasis. However, stressful environments (e.g., exposure to ultraviolet light, heat exposure, or nutrient stress) significantly increase ROS levels, which eventually results in significant damage to cell structures and ultimately all three types of PCD depending upon the ROS levels [31]. Somewhat higher levels of ROS can induce cell death through autophagy [32]. Very high ROS levels induce apoptosis through both the extrinsic and intrinsic pathways, and an even higher ROS levels can cause necrosis/necroptosis [33–37]. Considering all these, it was suspected that cis-khellactone may induce three types of PCD by regulating ROS production in cancer cells. To test this hypothesis, MCF7 and MDA-MB-231 cells were grown and treated with two different concentrations of cis-khellactone (5 and 10 μg/ml) in complete media for 24 or 48 h, and intracellular ROS content was evaluated using DCFH-DA. However, it was not possible to measure ROS after treatment with 10 μg/ml of cis-khellactone due to cell death. Therefore, further study was done with 5 μg/ml of cis-khellactone. The results showed that cis-khellactone treatment induced much higher levels of ROS in both cancer cell lines compared with the MCF10A normal cells (Figure 6A). ROS levels were about four or two times higher in MCF7 and MDA-MB-231 cells, respectively, compared with untreated control cells (Figure 6A). In contrast, ROS levels were found to be slightly decreased in MCF10A cells (Figure 6A). These results suggest that cis-khellactone treatment leads to the generation of much higher levels of ROS in cancer cells and may therefore provide stronger cell death signals to malignant cells compared with normal MCF10A cells. Next, the effect of cis-khellactone on MMP levels was also evaluated. Cis-khellactone treatment led to a rapid decrease in MMP in both cancer cell lines but not in normal cells (Figure 6B). In addition, when we checked the effect of cis-khellactone on cellular ROS and MMP levels in time- and concentration dependent ways, their changes were detected earlier than the cell death. Considering these data, we therefore conclude that cis-khellactone at first induces ROS generation and mitochondrial depolarization, which eventually stimulates three types of cell deaths.

To investigate how cis-khellactone affects cellular ROS levels and MMP, we checked the expression levels of many mitochondrial proteins that play vital roles in the initiation of PCD. Among them, BAX, BAK, and voltage-dependent anion channel 1 (VDAC1) proteins were found to be greatly affected by cis-khellactone treatment (Figure 6C). It is well known that cell death induced by many stimuli involves early mitochondrial activation, which is responsible for the subsequent disruption of mitochondrial respiratory chain (MRC) proteins, increased ROS production, loss of MMP, cytochrome c release, and ultimately cell death. Many different types of cellular stresses induce the release of cytochrome c from the mitochondria into the cytosol, which enhances caspases activation and promotes apoptosis and/or necrosis/necroptosis [38–39]. This process involves dynamic changes in the Bcl-2 family of proteins (*e.g.* BAX, BAK, Bcl-2, and Bcl-x_L_), opening of permeability transition channels, and the loss of MMP. Our data showed that cis-khellactone increased the level of BAX and BAK but not Bcl-2 and Bcl-x_L_ (Figure 6C). It is known that the oligomeric form of the pro-apoptotic protein BAX stimulates cytochrome c release. In addition, we found that levels of the VDAC1mitochondrial outer membrane protein also increased in mitochondria in response to cis-khellactone treatment. VDAC1 is known to be localized in the outer mitochondrial membrane and performs several important functions in the cell, including the regulation of cell deaths [40–42] VDAC1 is a key player in mitochondria-mediated apoptosis, participating in the release of mitochondrial pro-apoptotic proteins into the cytosol (e.g. cytochrome c, AIF, and Smac/DIABLO) [43]. Emerging research has revealed that overexpression and dysregulation of this channel could lead to apoptosis and a variety of diseases including cancer. In addition, VDAC1 oligomerization increases significantly with the induction of apoptosis [44].

Altogether, our data strongly suggest that cis-khellactone induces all three types of PCD in MCF7 and MDA-MB-231 cancer cells at least partly by regulating two important mitochondrial functions, induction of cellular ROS generation and suppression of MMP.

### *In vivo* confirmation of cis-khellactone as an anti-cancerous drug using xenograft model

**Figure 7:**
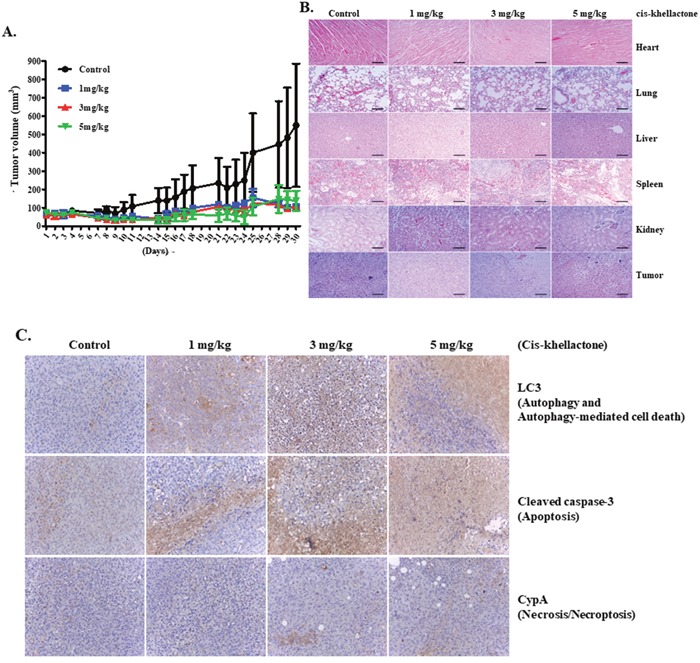
*In vivo* assessment of cis-khellactone anti-tumor activity in a murine model **(A)** Change in tumor volume after cis-khellactone treatment of a nude mouse. Tumor-bearing mice were injected with MDA-MB-231 cancer cells as mentioned in Materials and Methods. When tumors were approximately 50 to 100 mm^3^ in volume, mice in each treatment group were intravenously injected via the tail vein with cis-khellactone (at a dose of 1, 3, or 5 mg/kg) once every 3 days. Control groups received only normal saline (^*^P<0.01, *n* = 5, Student's *t* test). **(B)** Representative images of H&E staining of five major organs (heart, lung, liver, spleen, and kidney) and tumor. Mice were sacrificed at 30 days after the initial drug administration and tissue samples were immediately collected. Scale bar = 140 μm. **(C)** Induction of three PCD in xenograft mice tumor model with MDA-MB-231 cells were tested by employing Immunohistochemistry (IHC). Three PCD were checked with cleaved caspase 3 for apoptosis, LC3 for autophagic cell death, and CypA for necrosis/necroptosis.

To confirm the effectiveness of cis-khellactone as an anti-cancer drug *in vivo*, a xenograft model was used as described in Materials and Methods. *In vivo* anti-tumor activity of cis-khellactone was investigated in MDA-MB 231 tumor-bearing mice. When tumors were approximately 50 to 100 mm^3^ in volume, mice were treated with 1, 3, or 5 mg/kg cis-khellactone. The suppression of tumor growth was found to be inhibited by up to 85% in all cis-khellactone treatment groups compared with the untreated control group (P <0.01) (Figure 7A). No significant differences were found in tumor growth suppression among three group with the different dosage, suggesting that cis-khellactone can inhibit tumor growth even at small dosages. Very importantly, immunohistological analysis revealed that five representative organs from treated groups showed similar cytotoxic effects to that of the control group, suggesting that cis-khellactone affects only tumor cells but not normal cells (Figure 7B). In addition, we also checked whether cis-khellactone indeed can induce apoptotic, autophagic and necrotic cell deaths in xenograft mice tumor model with MDA-MB-231 cells, which showed the similar result with that of *in vitro* data. All three PCD were induced in MDA-MB-231 cells, relatively higher level of autophagy and apoptosis but lower level of necrosis/necroptosis (Figure 7C).

Taken together, our results suggest that cis-khellactone effectively inhibit tumor growth with minimal toxicity to normal tissues and organs.

## DISCUSSION

**Figure 8:**
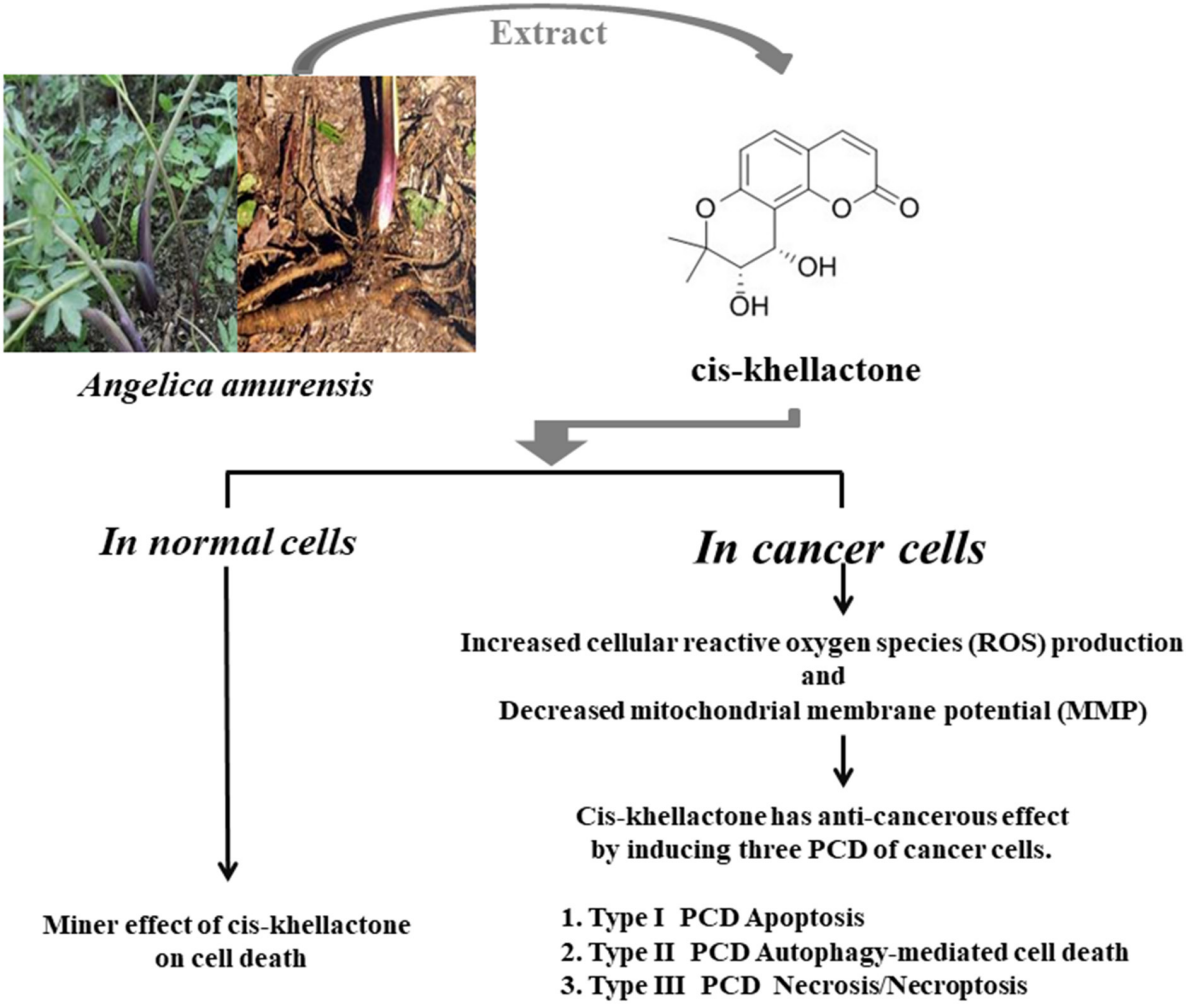
Schematic diagram showing how cis-khellactone from *Angelica amurensis* can induce three types of PCD (apoptosis, autophagy-mediated cell death, and necrosis/necroptosis) Cis-khellactone affected cell growth and viability of several different types of cancer cells in a time- and concentration-dependent manner. *In vitro* experiments showed that cis-khellactone can suppress cell growth and proliferation of cancer cells and inhibit migration at relatively low concentrations (2.5 and 5 μg/ml). Cis-khellactone can also induce three types of programmed cell death (apoptosis, autophagy-mediated cell death, and necrosis/necroptosis) at higher concentrations (10-20 μg/ml). Cis-khellactone induces autophagy, apoptosis, and necrosis/necroptosis in cancer cells in part by increasing ROS levels and decreasing MMP in cancer cells with minimal effects in normal cells. These findings were confirmed by *in vivo* experiments using xenografts.

Our ultimate goal is to develop a more effective anti-cancer drug with no toxicity to human. To overcome resistance of cancer cells to anti-cancer drugs, we have sought ways to induce several types of cell deaths simultaneously, using plant extracts with known medical properties. Our model shows how cis-khellactone functions as an anti-cancer agent in cancer cells (Figure 8). According to our results, cis-khellactone appears to possess time- and concentration-dependent anti-proliferation activity in all tested cancer cell lines. Interestingly, cis-khellactone also can induce three different types of PCD in malignant cells, including autophagy-mediated cell death at relatively low concentrations and apoptosis and necrosis/necroptosis at high concentrations. Importantly, cis-khellactone does not affect the viability of normal cells. In the effort of finding how cis-khellactone can induce three PCD, we at first checked two main mitochondrial functions, ROS levels and MMP because the redox balance is often impaired in cancer cells compared with normal cells. Our data revealed that cis-khellactone treatment led to the generation of much higher levels of ROS, signaling cell death in malignant cells but not in normal cells. In addition, its treatment also could lead to a rapid decline of MMP, rupture of the mitochondrial membrane, and ultimately cell death. Our data also suggest that cis-khellactone may be a good chemotherapeutic candidate for many different types of cancers, when used in combination with other cancer therapies.

In conclusion, our findings could contribute to the development of more effective but less toxic anti-cancer drugs. In addition, these results help lay the groundwork for the design and discovery of new anti-cancer drugs.

## MATERIALS AND METHODS

### Preparation of cis-khellactone from *Angelica amurensis*

A phytochemical study on *Angelica amurensis* led to the isolation and structural characterization of cis-khellactone (PubChem CID: 11097348). The rhizomes of *Angelica amurensis* (1.5 kg) were re-dried and freeze-dried for 2 days and subjected to extraction at room temperature with MeOH three times (5 L each) over one week. The methanolic extract was filtered through a Buchner funnel using Whatman No. 1 filter paper. The methanolic residue (211.4 g) was diluted with water and partitioned against chloroform (38.3 g). The chloroform-soluble extracts were chromatographed on silica gel (10 × 40 cm, 230–400 mesh) that acted as the stationary phase with a solvent system [hexane (5 L), hexane-CH_2_Cl_2_ (1 : 1 v/v, 5 L), CH_2_Cl_2_ (5 L), CH_2_Cl_2_MeOH (19 : 1 v/v, 5 L), CH_2_Cl_2_-MeOH (15 : 5 v/v, 5 L), CH_2_Cl_2_-MeOH (1 : 1 v/v, 5 L), CH_2_Cl_2_-MeOH (5 : 15 v/v, 5 L), CH_2_Cl_2_-MeOH (1 : 19 v/v, 5 L), MeOH (2 L)] to afford 9 pooled fractions (Fr:01–Fr:09). Fraction F06 [eluted with CH_2_Cl_2_-MeOH (15:5 v/v); 7.4 g] was chromatographed over silica gel (5 × 40 cm, 230–400 mesh; n-hexane-EtOAc gradient from 20 : 1 to 1 : 1 v/v, final 100% MeOH) resulting in 14 sub fractions (Fr:06–01 to Fr:06–14) on the basis of TLC profiles. By comparing the spectral data (^1^H NMR (400 MHz, CDCl_3_) δ: 6.27 (*d*, 9.5), 7.67 (*d*, 9.5), 7.34 (*d*, 8.7), 6.81 (*d*, 8.7), 3.89 (*d*, 5.0), 5.23 (*d*, 5.0), 1.42 (*s*), 1.48 (*s*)) with previous report [45], the major compound was identified as cis-khellactone (Supplementary Figure 2).

### Cell lines, cell culture, and treatment of cancer cells with cis-khellactone

Nine breast cancer cell lines (MCF7, MDA-MB-231, BT20, BT549, T47D, SKBR3, MDA-MB-453, HS578T, and MDA-MB-468), two colon cancer cell lines (HCT116 and HT-29), two cervical cancer cell lines (HeLa and SiHa), human embryonic kidney cell lines (HEK293T and HEK293), and normal human and mouse cell lines (MEF and NIH3T3) were cultured in Dulbecco's Modified Eagle's Medium (DMEM, WelGENE Inc., Korea) supplemented with 10% fetal bovine serum (FBS) (Gibco BRL, USA) and 1% Antibiotic-Antimycotic solution (Gibco BRL, Cat#15240-062, USA). A normal human MCF10A mammary epithelial cell line was grown in DMEM/F-12 medium (Gibco BRL, Cat#11330-032, USA) supplemented with 20 ng/ml of epidermal growth factor (EGF) (Sigma-Aldrich, Cat#E9644, USA), 100 ng/ml of cholera toxin (Sigma-Aldrich, Cat#C-8052, USA), 10 μg/ml of insulin (Sigma-Aldrich, Cat#I-9278, USA), 0.5 mg/ml of hydrocortisone (Sigma-Aldrich, Cat#H-0888, USA), 5% horse serum (Invitrogen, Cat#16050-122, Korea), and 1% Antibiotic-Antimycotic solution. All cells were cultured at 37°C in a humidified atmosphere composed of 95% air and 5% CO_2_. The SiHa cell line was obtained from the Korean Cell Line Bank (KCLB #30035); other cell lines were purchased from the American Type Culture Collection (ATCC). All cell lines were treated with either DMSO alone as a control or with 1, 2.5, 5, 10, or 20 μg/ml of cis-khellactone for the indicated times.

### Cell viability analysis

Cell viability was analyzed using the Cell Viability, Proliferation & Cytotoxicity Assay Kit (EZ-CYTOX, Cat# EZ-3000, DoGen, Korea) according to the manufacturer's instructions, and experiments were conducted in triplicate. Briefly, cells (2 × 10^4^/well) were grown in 96-well plates containing DMEM medium and exposed to indicated concentrations of cis-khellactone. After 24 or 48 h, 10 μl of CCT was added to the medium and cells were incubated in a CO_2_ incubator at 37 °C for 30 min. Cell viability was assessed by measuring the absorbance at 450 nm with a Gemini XPA Microplate Reader. The number of viable cells was also evaluated using a trypan blue staining method.

### Analysis of autophagy, apoptosis, and necrosis/necroptosis

Autophagy, apoptosis, and necrosis/necroptosis were analyzed by Western blot with the following corresponding biomarkers or regulatory proteins: p62 and LC3 for autophagy; PARP for apoptosis; CypA for necrosis/necroptosis. Necrosis/necroptosis was evaluated by measuring levels of the extracellular CypA biomarker protein, which is released from necroptotic cells [46].

### Western blotting

Cells were centrifuged, washed in ice-cold phosphate-buffered saline (PBS), and then lysed in radio immunoprecipitation assay (RIPA) lysis buffer. The amount of protein was quantified using a protein assay kit (Bio-Rad, Korea). Each sample was subjected to SDS-PAGE and transferred to an Immobilon Transfer Membrane (Millipore, Cat#IPVH00010). The filter was incubated with each corresponding antibody, and immune-detection was carried out using the PowerOpti-ECL Western blotting detection reagent (Bio-Rad, Korea). The following antibodies were used in this study: PARP (Cell Signaling, Cat#9542S), BAX (Santa Cruz Biotechnology, Cat. sc7480), BAK (Cell signaling, Cat. 3814S), cyclophilin A (CypA)(Enzo Life Sciences, BML-SA296), LC3 (Enzo Life Sciences, ALX-803-082), p62/SQSTM1 (Cell Signaling, Cat#5114), VDAC1 (Santa Cruz Biotechnology, Cat. sc-32063), HSP60 (Santa Cruz Biotechnology, Cat. sc13966), β-actin (Santa Cruz Biotechnology, Cat. sc-47778), and γ-tubulin (Santa Cruz Biotechnology, Cat. sc-7396). The result of Western blot was quantified by using ImageJ program (Image Processing and Analysis in Java, wsr@nih.gov).

### Measurement of reactive oxygen species (ROS)

Cells were treated with cis-khellactone for 24 h, collected, washed with PBS, and centrifuged in 15 ml conical tubes at 1,000 rpm for 2 min. Approximately ~5 × 10^4^ cells in 50 μl of PBS were transferred onto 96-well plates and 50 μl of 200 μM 2′,7′dichlorodihydro-fluorescein diacetate (DCFH-DA) was added with multichannel-pipettes (PIPETMAN, Gilson) into each well to achieve a final DCFH-DA concentration of 100 μM. ROS levels were detected by measuring the amount of fluorescence from DCFH-DA at an excitation wavelength of 485 nm and an emission wavelength of 535 nm every 5 min for 30 min with a Gemini XPA Microplate Reader.

### Measurement of mitochondrial membrane potential (MMP)

MMP was determined using a Mito-ID Membrane Potential Cytotoxicity Kit (Enzo Life Sciences, Farmingdale, NY, USA). Briefly, cells were cultured in 96 well tissue culture dishes (SPL Life Science, Korea) with DMEM media, followed by cis-khellactone treatment for 2 h. Mito-ID Membrane Potential Dye Loading Solution was added to each well, followed by 30 min of incubation at room temperature. Mitochondria produce an orange fluorescence signal following aggregation of the Mito-ID dye. MMP was assessed by measuring the resulting fluorescence with a Gemini XPA Microplate Reader, using an excitation wavelength of 480 nm and an emission wavelength of 590 nm.

### Preparation of mitochondrial fractions

Cells were centrifuged at 2,000 rpm for 5 min, washed in ice-cold PBS, and resuspended with hypotonic lysis buffer (220 mM mannitol, 10 mM HEPES, 2.5 mM PO_4_H_2_ K, 1 mM EDTA, 68 mM sucrose, and 1 mM PMSF). They were kept on ice for 60~90 min and then centrifuged at 1,000 × *g* for 5 min at 4°C. The pellets were resuspended in mitochondrial fraction buffer and pipetted every 10 min during the incubation on ice. After removing cellular debris by centrifugation at 1,500~2,000 × *g* at 4 °C for 5 min, the supernatant was transferred to a fresh tube and centrifuged at 10,000~14,000 ×*g* for 5 min at 4°C. At this point, the supernatant and pellet represented the cytosolic and mitochondrial fractions, respectively. For better purity, the supernatant was centrifuged again following the same protocol. Pellets were resuspended with RIPA buffer and used for Western blot analysis as described in Materials and Methods. Total protein, as well as cytosolic and mitochondria fractions, were prepared for Western blot analysis.

### *In vivo* anti-tumor activity

The anti-tumor activity of cis-khellactone was evaluated in MDA-MB-231 tumor-bearing mice. Twenty mice were divided into one control and three treatment groups, which received different concentrations of cis-khellactone. There were five mice in each group. When tumors were approximately 50 to 100 mm^3^ in size, mice in the treatment groups were intravenously injected via the tail vein with cis-khellactone (at a dose of 1, 3, or 5 mg/kg) once every 3 days. Control groups were injected with normal saline. Tumor size was measured daily for 30 days and the tumor volumes were calculated as a × b^2^/2, where “a” and “b” indicate the largest and smallest diameters, respectively. Mice were sacrificed 30 days after drug administration. Tissue samples, including heart, lung, liver, spleen, and kidney, were immediately collected for pathological analysis.

### Hematoxylin and eosin (H&E) staining

H&E staining was conducted following standard procedures. Briefly, after nude mice with MDA-MB 231 xenografts were sacrificed, tissue from the heart, lung, liver, spleen, kidney and tumor were fixed in 4% paraformaldehyde for 24 h, washed in PBS buffer, and then embedded in paraffin. Tissue samples were then sliced into 5 μm sections and stained with H&E.

### Immunohistochemistry (IHC)

Formalin-fixed and paraffin-embedded sections of 5-mm thickness were dried at 60°C for 1 h, deparaffinized in xylene, rehydrated through graded alcohols, and immersed for 15 min in PBS buffer. For antigen retrieval, the sections were microwaved in 0.01 M citrate buffer (pH 6.0) for 20 min. After the microwave pretreatment, the endogenous peroxidase activity was blocked with 3% hydrogen peroxidase in methanol for 10 min. The sections were incubated for 20 min with normal horse serum to block nonspecific staining. The sections were sequentially incubated with a primary antibody to cleaved caspase-3 (1:500, Cell signaling, Cat#9661S), LC3 (1:500, Enzo Life Sciences, ALX-803-082), CypA (1:500, Enzo Life Sciences, BML-SA296), a biotinylated secondary antibody (DAKO envisioin kit), and 0.06% diaminobenzidine containing 0.01% hydrogen peroxidase. Finally, the sections were counterstained with hematoxylin.

### Statistical analysis

Statistical analysis was performed with Student's *t*-test using SPSS software (SPSS Inc., Chicago, IL, USA). Data from triplicate experiments were used for each analysis, and *P* < 0.05 was considered statistically significant.

## SUPPLEMENTARY MATERIALS FIGURES


