# Supplementary material for: Anti-cancerous effect of cis-khellactone from Angelica amurensis through the induction of three programmed cell deaths
Source: Oncotarget. 2018 Mar 30;9(24):16744–57. doi: 10.18632/oncotarget.24686 (PMC5908283; doi:10.18632/oncotarget.24686)
Supplement: Supplementary file 1 [file oncotarget-09-16744-s001.pdf]

Anti-cancerous effect of cis-khellactone from *Angelica amurensis* through the induction of three programmed cell deaths

SUPPLEMENTARY MATERIALS

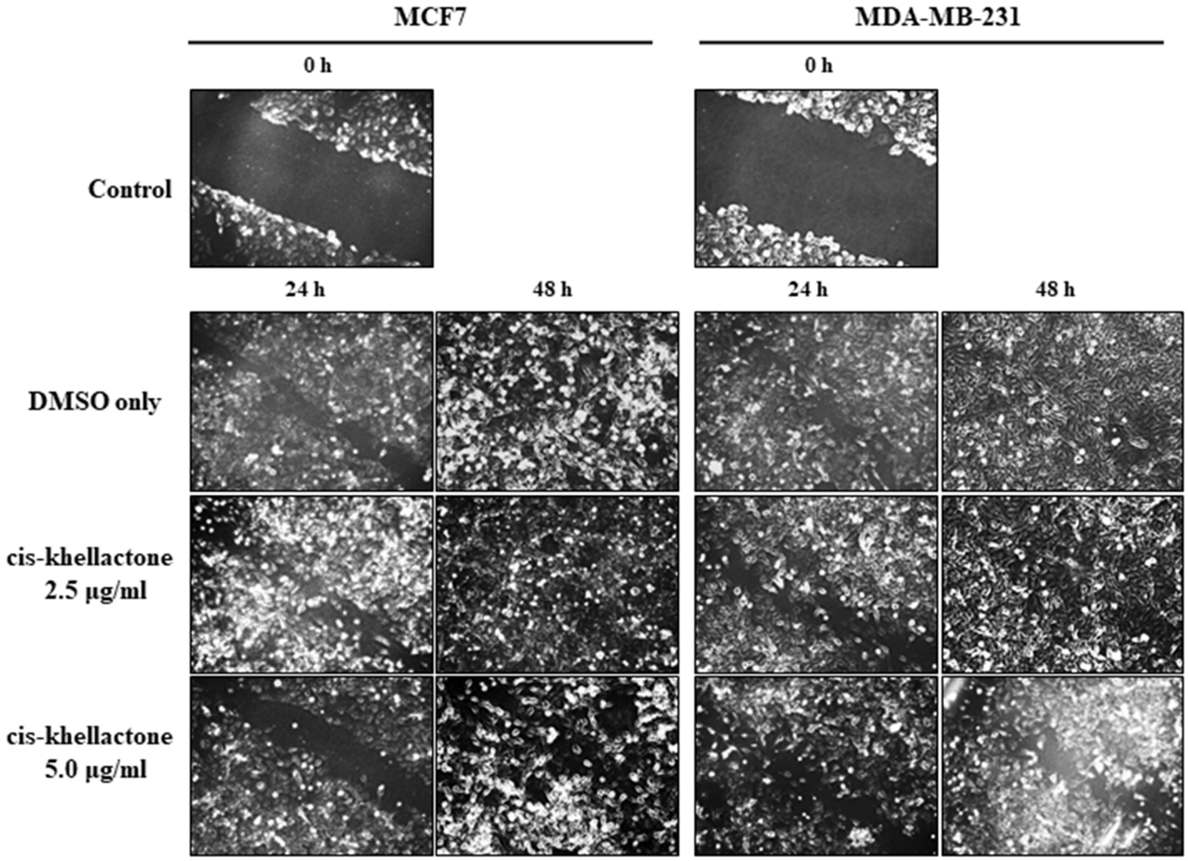

Supplementary Figure 1: Inhibitory role of cis-khellactone on the migration of MCF7 and MDA-MB-231 cells. MCF7 and MDA-MB-231 cells were plated onto 60-mm tissue culture dishes and allowed to form a confluent monolayer. The cells were then treated with 2.5, 5, or 10 µg/ml cis-khellactone for the indicated times, and morphologic changes were photographed under a microscope. Wounds were introduced in the monolayer by scratching with the tip of a plastic micropipette, and the migration rate was quantified by photographing the plates at indicated intervals until the wound size decreased (scale bar = 100 µm).

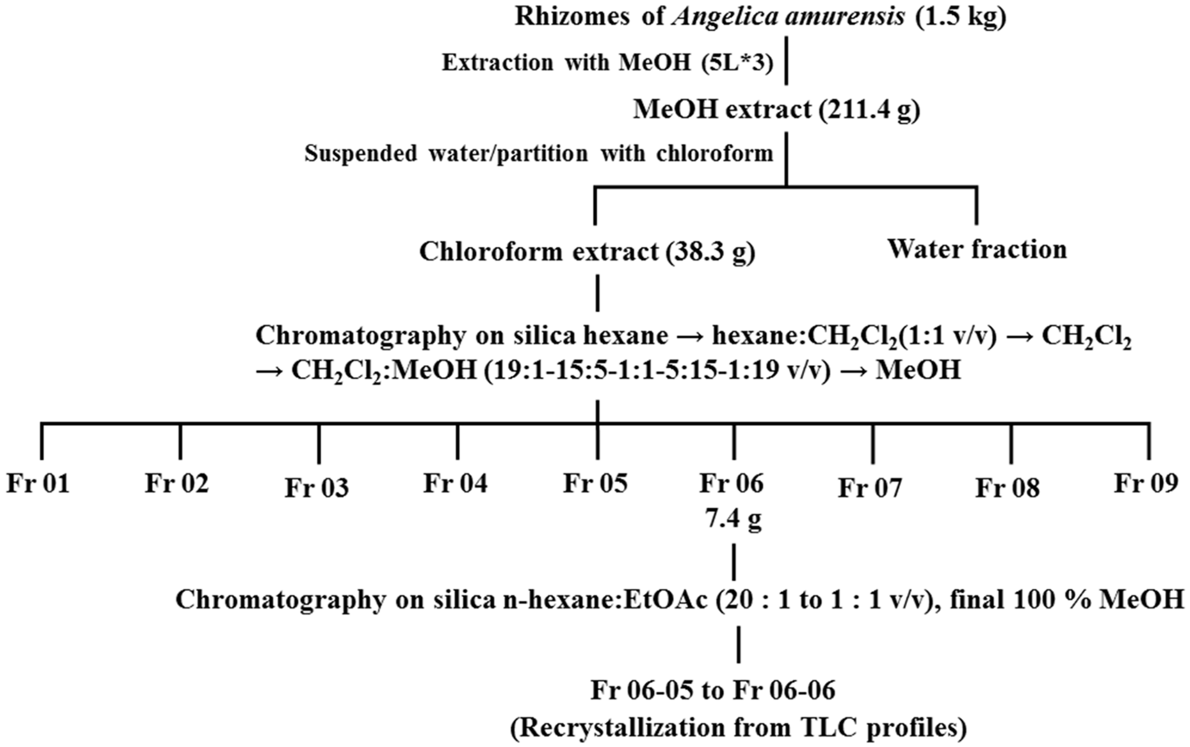

Supplementary Figure 2: Separation of cis-khellactone from *Angelica amurensis*.
